# Estimated Cardiorespiratory Fitness and Risk of Incident Frailty in Middle-Aged and Older Adults: A Cross-National Longitudinal Cohort Study

**DOI:** 10.3390/healthcare14091169

**Published:** 2026-04-27

**Authors:** Haoqi Yan, Jingjing Liang, Haozhe Huang, Ming Chen, Cheng Hu, Leyan Wang, Wei Li, Botao Wu, Guantong Fang, Juan Ge

**Affiliations:** Department of Respiratory and Critical Care Medicine, The Second Affiliated Hospital of Xi’an Jiaotong University, Xi’an 710004, China; 1473688872@stu.xjtu.edu.cn (H.Y.); 2234122479@stu.xjtu.edu.cn (J.L.); hzhuang@stu.xjtu.edu.cn (H.H.); 13027255686@163.com (M.C.); 2234224093@stu.xjtu.edu.cn (C.H.); katie_wang@stu.xjtu.edu.cn (L.W.); liweihuxi@163.com (W.L.); 15637395207@163.com (B.W.); 2234112445@stu.xjtu.edu.cn (G.F.)

**Keywords:** estimated cardiorespiratory fitness, incident frailty, frailty index, healthy ageing, cohort study

## Abstract

(1) Background: Frailty is a major geriatric syndrome associated with adverse health outcomes, while direct assessment of cardiorespiratory fitness (CRF) is often impractical in routine clinical settings. This study investigated the association between estimated cardiorespiratory fitness (eCRF) and incident frailty in middle-aged and older adults from three nationally representative aging cohorts. (2) Methods: We analyzed longitudinal data from the Health and Retirement Study (HRS; 2006–2020) in the United States, the English Longitudinal Study of Ageing (ELSA; 2004–2018) in England, and the China Health and Retirement Longitudinal Study (CHARLS; 2011–2018) in China. Participants aged 50 years or older were included. eCRF was calculated using validated sex-specific non-exercise algorithms. Frailty was assessed using a 30-item Frailty Index (FI), and incident frailty was defined as FI ≥ 0.25. Cox proportional hazards models were used to evaluate the association between baseline eCRF and incident frailty. (3) Results: A total of 8152 participants (3982 women and 4170 men) were included in the longitudinal analysis. Each 1-SD increase in eCRF was associated with a lower risk of incident frailty in HRS (HR = 0.60, 95% CI: 0.54–0.68), ELSA (HR = 0.54, 95% CI: 0.46–0.64), and CHARLS (HR = 0.74, 95% CI: 0.63–0.87). Compared with the low-eCRF group, the moderate- and high-eCRF groups had progressively lower risks of incident frailty across all three cohorts, indicating a graded inverse dose–response relationship. Findings were generally consistent across subgroup and sensitivity analyses. (4) Conclusions: Higher eCRF was associated with a lower risk of incident frailty among middle-aged and older adults across three nationally representative cohorts. As an accessible, non-invasive metric, eCRF may be useful for identifying individuals at elevated risk of incident frailty.

## 1. Introduction

As population aging accelerates, frailty has become an increasingly important global public health concern, with implications for both clinical practice and public health policy. Moreover, the burden of frailty is expected to continue increasing [1]. Characterized as a common geriatric syndrome, frailty manifests primarily through diminished multisystem physiological capacity and heightened vulnerability to stressor exposure [2]. The Frailty Index (FI), developed by Rockwood and colleagues, is widely used to assess frailty. This metric quantifies the proportion of accumulated age-associated health deficits [3]. Furthermore, previous studies have linked frailty to multiple adverse health outcomes, including mortality [4,5,6], falls [7], depressive symptoms [8], cardiovascular disease (CVD) [9], and chronic liver disease [10]. Because frailty is a dynamic and potentially reversible state, identifying its determinants is important [1,9,11,12,13]. Although the association between frailty and cardiovascular health has been increasingly recognized, whether cardiovascular health status influences the incidence of frailty remains insufficiently understood.

Cardiorespiratory fitness (CRF) reflects the overall efficiency of oxygen delivery and utilization during physical activity, serving as an indicator of general health status. Substantial evidence has demonstrated inverse associations between CRF and the risk of type 2 diabetes [14], non-fatal CVD events [15], and mortality [15,16,17]. Unlike conventional risk indicators, CRF assessment is not routinely performed in clinical practice because precise measurement requires exercise testing conducted by trained personnel using specialized equipment. Consequently, several non-exercise algorithms have been developed to estimate CRF using routinely available health metrics in clinical and healthcare settings. Estimated CRF (eCRF) has shown acceptable concurrent validity compared with the CRF measured by exercise testing [15]. eCRF is capable of predicting long-term mortality and CVD risk, with accuracy similar to that obtained through directly measured CRF [18,19]. An earlier American cohort investigation suggested that higher CRF was associated with a slower increase in FI over time [20], indicating that CRF may be a potentially modifiable factor related to frailty. Nevertheless, the predictive capacity of eCRF for incident frailty requires further investigation.

Frailty affects millions of older adults worldwide, making it a major public health concern. Therefore, clarifying the association between eCRF and frailty may help inform strategies to reduce the burden of frailty in aging populations. Against this background, we used data from three well-established prospective cohorts, the Health and Retirement Study (HRS), the English Longitudinal Study of Ageing (ELSA), and the China Health and Retirement Longitudinal Study (CHARLS), to investigate the association between eCRF and incident frailty in middle-aged and older adults. By leveraging these three nationally representative ageing cohorts, we also aimed to examine whether this association was consistent across diverse population settings.

## 2. Materials and Methods

### 2.1. Study Design and Population

Our investigation analyzed longitudinal datasets from three major aging studies conducted across different regions: HRS in the United States [21], ELSA in England [22], and CHARLS in China [23]. These studies specifically enrolled participants aged 50 years or above. Comprehensive methodological profiles for each cohort are available in the Supplemental Methods. To ensure international comparability, the ELSA and CHARLS projects adopted design elements and procedures consistent with the HRS methodology. Each survey was conducted under the supervision of leading research institutions in the respective countries. The research protocol received ethical approval from the institutional review boards at the University of Michigan, the London Multicenter Research Ethics Committee, and Peking University. All procedures followed the principles of the Declaration of Helsinki, and written informed consent was obtained from all participants.

The use of broadly comparable data collection procedures and assessment tools supports multinational comparative research using these datasets. The consistency in methodological approaches has been previously affirmed in terms of both reliability and analytical validity [9,24]. For the current analysis, anthropometric measures from specific survey waves were included: HRS data covering 2006 to 2020 (waves 8–15), ELSA from 2004 to 2018 (waves 2–9), and CHARLS between 2011 and 2018 (waves 1–4). The baseline assessment corresponded to wave 8 (2006) for HRS, wave 2 (2004) for ELSA, and wave 1 (2011) for CHARLS.

### 2.2. Assessment of eCRF

eCRF, expressed in metabolic equivalents (METs; 1 MET equals 3.5 mL O_2_/kg/min), is derived from factors including sex, age, body mass index (BMI), waist circumference (WC), resting heart rate (rHR), physical activity level, and smoking habits. The sex-specific non-exercise prediction models were originally developed in large Western cohorts [25] and have since been validated across multiethnic populations, including Chinese adults. Recent cross-national research using harmonized HRS, ELSA, and CHARLS data confirmed the model’s reliability and cross-cultural comparability [24]. Accordingly, the same equations were adopted in this study to ensure methodological consistency and to facilitate international comparability of findings. Sex-specific prediction models, developed from longitudinal studies, enhance the accuracy and practicality of eCRF estimation [19,25], improving its feasibility for research and clinical use. The equations were as follows: Men: eCRF = 21.2870 + (age × 0.1654) − (age^2^ × 0.0023) − (BMI × 0.2318) − (WC × 0.0337) − (rHR × 0.0390) + (moderate-to-vigorous physical activity × 0.6351) − (smoking × 0.4263); Women: eCRF = 14.7873 + (age × 0.1159) − (age^2^ × 0.0017) − (BMI × 0.1534) − (WC × 0.0085) − (rHR × 0.0364) + (moderate-to-vigorous physical activity × 0.5987) − (smoking × 0.2994).

At baseline, participants’ age was recorded in years. BMI was computed as the ratio of body weight in kilograms to height in meters squared. WC was assessed through a horizontal measurement taken from the top of the right iliac crest to the umbilicus and was recorded in cm. rHR was evaluated based on pulse measurements taken per minute. Physical activity level was categorized using questionnaire data on self-reported exercise intensity and frequency: Individuals were assigned a value of 1 if they reported moderate-to-vigorous physical activity at least once per week, and 0 if they engaged in it on a less frequent basis. Participants were classified according to smoking status as current smokers (assigned a value of 1) or non-smokers (assigned a value of 0).

Using age- and sex-adjusted eCRF quintile distributions [26,27,28], the study population was stratified into three distinct fitness categories: low (representing the first quintile), moderate (encompassing the second and third quintiles), and high (comprising the fourth and fifth quintiles).

### 2.3. Assessment of Frailty

Frailty was assessed using a FI, which was derived from the accumulation of multiple age-associated health deficits. Data from the HRS, ELSA, and CHARLS surveys were utilized to identify 30 items for FI construction [9,29]. To ensure cross-cohort comparability, FI construction followed the same standardized deficit-accumulation framework across the three cohorts, and the full item list and coding details are provided in Supplementary Table S1. These items covered multiple health domains, including medical conditions, symptoms, functional limitations, motor function, depressive symptoms, and cognitive function. With the exception of item 30, all variables were transformed into dichotomous outcomes (0 or 1) according to predefined criteria. In this coding system, a value of 0 indicated the absence of a deficit, whereas a value of 1 indicated its presence. Item 30, representing cognitive performance, was retained as a continuous measure on a scale from 0 to 1, with elevated scores corresponding to more severe cognitive impairment.

The FI was derived by summing all component scores and dividing this total by the number of assessed items, producing a continuous metric ranging from 0 to 1. An elevated FI value corresponds to a more severe state of frailty. For the purpose of this analysis, a threshold of 0.25 was applied to classify the study participants into two distinct states: a non-frail state (FI < 0.25) and a frail state (FI ≥ 0.25) [1,5,29].

### 2.4. Covariates

Data on key covariates, including participants’ demographic details, were collected via questionnaires within the respective studies. The set of covariates included sociodemographic characteristics such as age, sex, marital status, education level, employment situation, and household composition, including co-residence with children. Health-related behaviors, including smoking, alcohol intake, and physical activity, were also considered. In addition, anthropometric measures (BMI, WC, and rHR) and medical histories of specific conditions, including cancer, pulmonary disease, and CVD, were incorporated. To ensure comparability across the three cohorts, relevant categorical variables were harmonized. Specifically, common categories were created across cohorts for education, marital status, co-residence with children, smoking status, alcohol consumption, and physical activity to support consistent cross-cohort comparisons, while detailed harmonization procedures are described in the Supplemental Methods. In addition to summarizing missing values for certain covariates (Supplementary Table S2), the Supplemental Methods provide further details on the covariates used in this study. Although most variables were complete, several in ELSA showed varying degrees of missingness, including education (8.60%), employment (59.51%), living with children (14.04%), and drinking status (7.02%). Therefore, the primary analyses were conducted using a complete-case approach. Multiple imputation was additionally performed as a sensitivity analysis to evaluate the robustness of the findings to missing data.

### 2.5. Statistical Analysis

In descriptive analyses, continuous measures were summarized using either means with standard deviations (SD) or medians with interquartile ranges (IQR), while categorical measures were expressed as numbers with percentages. As an additional descriptive analysis, we compared the individual components used to estimate eCRF between frail and non-frail participants at baseline within each cohort. The cumulative incidence of frailty was visualized through Kaplan–Meier curves, with group differences assessed via log-rank testing. Cross-sectional associations between eCRF and frailty status at baseline were evaluated using logistic regression. To examine longitudinal associations between eCRF and incident frailty over follow-up, we applied Cox proportional hazards models to derive hazard ratios (HR) and corresponding 95% confidence intervals (CI). Frailty was assessed at discrete follow-up waves rather than continuously. Therefore, for participants who developed incident frailty, event time was defined as the follow-up time corresponding to the first wave at which frailty was observed. Participants who did not develop incident frailty were censored at the last follow-up wave with a non-missing frailty assessment. The proportional hazards assumption in the Cox models was verified through Schoenfeld residual testing, incorporating both global and covariate-specific examinations. This assumption was considered satisfied when statistical tests yielded non-significant results (*p* > 0.05). Three statistical models were developed for both cross-sectional and longitudinal analyses: Model 1 contained no covariates; Model 2 incorporated age and sex adjustments; Model 3 further included education, employment situation, marital status, co-residence with children, and alcohol consumption. For clarity, the main cross-sectional results are presented using the fully adjusted model. Participants were stratified into low, moderate, and high eCRF levels according to eCRF values, with the lowest eCRF group serving as the reference. Potential nonlinear relationships between eCRF and incident frailty were explored using restricted cubic splines (RCS) within Cox models, with comprehensive adjustment for all Model 3 covariates and the reference point established at the eCRF level where HR = 1.

To evaluate potential effect heterogeneity, we conducted stratified analyses across various demographic and clinical characteristics to examine whether these factors modified the association of eCRF with incident frailty. The stratification variables included age (<70 years or ≥70 years), sex (female or male), education (below high school, high school or above high school), marital status (married and partnered or unmarried and others), co-residence with children (no or yes), physical activity (moderate-to-vigorous or others), BMI (normal weight, overweight, or obesity), smoking status (current, former, or never), and alcohol consumption (no or yes). To test for potential interaction effects, we utilized likelihood ratio tests to examine the significance of the relevant terms. Because some stratification variables, particularly physical activity and smoking, are also components of the eCRF algorithm, these subgroup analyses were considered exploratory and should be interpreted cautiously.

Five complementary sensitivity assessments were conducted to verify the stability of our main results. First, to mitigate potential reverse causality, we excluded participants with baseline frailty at the initial follow-up wave. Second, to reduce confounding from pre-existing health conditions, including cancer, lung disease, or CVD, these participants were excluded to examine whether the association between eCRF and incident frailty was robust to pre-existing disease. Third, to evaluate potential influences of complete-case analysis on our conclusions, we eliminated subjects with incomplete data for any variable incorporated in the logistic or Cox proportional hazards regression models. Fourth, to counter potential selection bias due to missing data, we applied multiple imputation via chained equations (MICE), creating 20 imputed datasets. These analyses were intended to assess the robustness of the complete-case results rather than to replace the primary analytic sample. Fifth, to examine whether the observed association was independent of baseline deficit burden among participants who were non-frail at baseline, we additionally adjusted the Cox models for baseline FI as a continuous variable. All statistical computations were performed using R software version 4.5.1. A two-sided *p*-value of less than 0.05 was established for statistical significance.

## 3. Results

### 3.1. Baseline Characteristics of the Study Population

**Figure 1:**
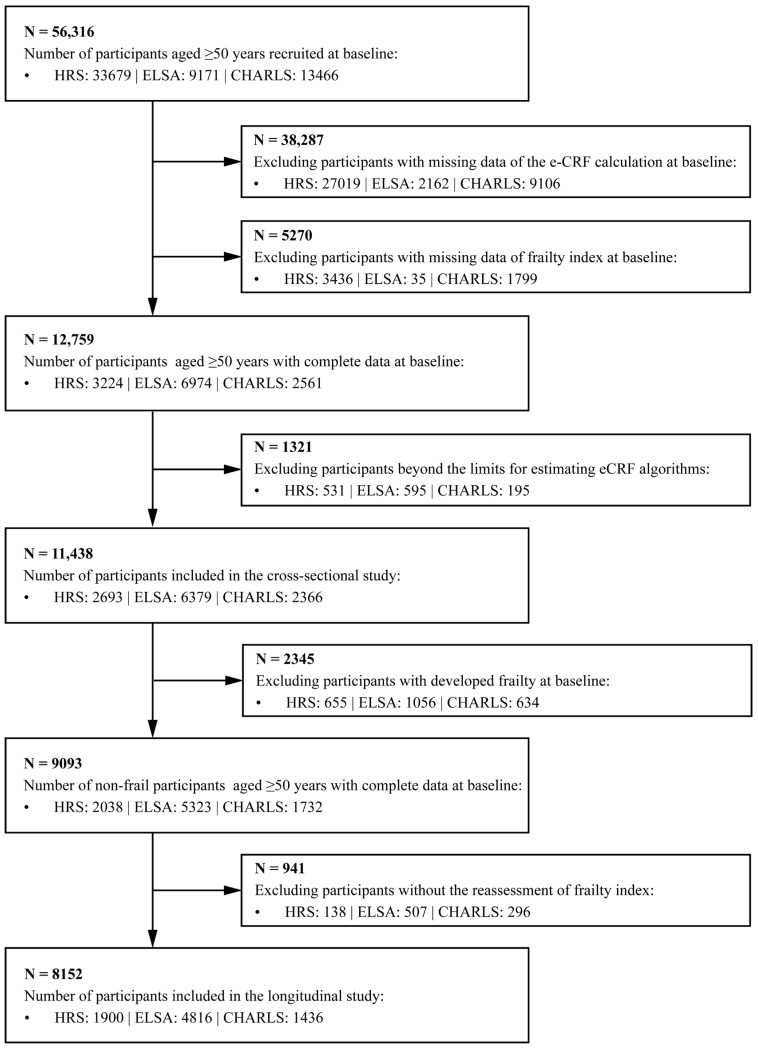
Flow chart of the study.

From an initial cohort comprising 56,316 participants with a baseline age of ≥50 years, a series of pre-defined exclusion criteria were implemented. We first excluded 38,287 individuals with missing data for the eCRF calculation, followed by an additional 5270 with missing FI data at baseline. After the additional exclusion of 1321 participants who did not meet the requirements for valid eCRF estimation, the cross-sectional baseline analytic sample comprised 11,438 participants, and their baseline characteristics are presented in Supplementary Table S3. For the longitudinal analysis on incident frailty, we further excluded 2345 participants who were frail at baseline and 941 individuals who did not undergo a reassessment of the FI during follow-up, yielding a final analytical sample of 8152 participants. Baseline characteristics of participants included in the longitudinal analysis are presented in Table 1. The largest reductions in sample size were attributable to missing information required for eCRF estimation and FI construction. Figure 1 depicts the participant selection process. To improve transparency regarding possible selection bias, we compared the demographic and health profiles of participants included in the analytical sample with those who were excluded. Compared with included participants, excluded individuals generally had lower eCRF levels, higher frailty burden, and a less favorable health profile (Supplementary Table S4).

We compared demographic and baseline health profiles across cohorts, including mean age, sex distribution, eCRF values, and FI. As detailed in Table 1, the mean age of participants was 71.54 years in the HRS cohort, 63.87 years in ELSA, and 60.28 years in the CHARLS population. The proportion of male participants was 49.37% in HRS, 48.73% in ELSA, and 61.63% in CHARLS. Mean baseline eCRF values were 9.33, 9.19, and 10.78 METs, respectively (Supplementary Figure S1). Median frailty indices were 0.12, 0.08, and 0.13, in the same order. All baseline characteristics differed significantly among cohorts (all *p* < 0.001).

Participants in the HRS cohort were generally older and more highly educated, with a majority reporting married or partnered status. This group exhibited higher rates of alcohol use and moderate-to-vigorous physical activity, along with elevated BMI, WC, and rHR. The prevalence of cancer, lung disease, and cardiovascular conditions was relatively high, and the FI was moderate. The ELSA cohort demonstrated lower eCRF levels, higher proportions of unmarried or non-partnered individuals, and more frequent smoking and alcohol use. Waist circumference was also elevated, though cancer and CVD were less common than in HRS. This group had the lowest FI among the three cohorts. In contrast, the CHARLS cohort had the youngest mean age and the highest percentage of males. Most participants were married or in a partnered relationship and lived with their children. Current smoking was common, educational attainment was relatively low, and engagement in moderate or vigorous physical activity was minimal. Several anthropometric measures and rates of chronic conditions demonstrated lower values relative to the remaining cohorts, while the FI was slightly higher than that of ELSA.

To improve the interpretability of eCRF as a composite measure, we additionally compared the individual components used to estimate eCRF between frail and non-frail participants at baseline in each cohort. Across the three cohorts, frail participants generally had lower continuous eCRF levels, lower participation in moderate-to-vigorous physical activity, and higher BMI, waist circumference, and resting heart rate than non-frail participants, while smoking patterns showed some heterogeneity across cohorts (Supplementary Table S5). The distribution of baseline characteristics according to eCRF categories is presented in Supplementary Tables S6–S8. In all study populations, higher eCRF was consistently associated with higher educational attainment, greater engagement in moderate-to-vigorous physical activity, and improved values for BMI, WC, rHR, and FI. More frequent alcohol consumption was observed among high eCRF participants in both HRS and ELSA; however, this pattern was not evident in CHARLS. With respect to disease distribution, higher eCRF was associated with a higher prevalence of CVD in HRS but a lower prevalence in CHARLS. No notable variations were detected in cancer or pulmonary disease occurrence among the three cohorts.

### 3.2. Cross-Sectional Associations Between eCRF and Baseline Frailty

Table 2 presents the fully adjusted odds ratios for the cross-sectional associations between eCRF and frailty status at baseline. Relative to the low eCRF reference category, moderate eCRF was associated with significantly lower odds of frailty across all three cohorts: HRS (OR = 0.40, 95% CI: 0.32–0.50, *p* < 0.001), ELSA (OR = 0.42, 95% CI: 0.25–0.73, *p* = 0.002), and CHARLS (OR = 0.61, 95% CI: 0.48–0.79, *p* < 0.001). Higher eCRF levels were associated with lower odds of frailty in both HRS (OR = 0.30, 95% CI: 0.23–0.38, *p* < 0.001) and ELSA (OR = 0.11, 95% CI: 0.05–0.24, *p* < 0.001), and a similar inverse association was observed in CHARLS (OR = 0.56, 95% CI: 0.43–0.72, *p* < 0.001).

### 3.3. Relationships Between Baseline eCRF and Incident Frailty

**Figure 2:**
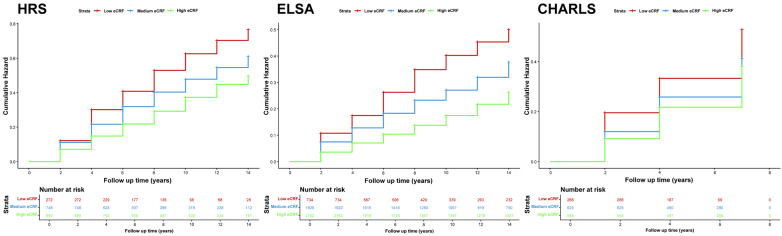
Kaplan–Meier curves for the cumulative incidence of frailty. Abbreviations: HRS, Health and Retirement Study; ELSA, English Longitudinal Study of Ageing; CHARLS, China Health and Retirement Longitudinal Study; eCRF, estimated cardiorespiratory fitness.

In the longitudinal study, 1900 participants in HRS, 4816 in ELSA, and 1436 in CHARLS were included. Kaplan–Meier curves across eCRF categories showed a consistent stepwise divergence in the cumulative hazard of frailty. Across all three cohorts, higher eCRF levels corresponded to a lower cumulative hazard of incident frailty. Specifically, the low eCRF group exhibited a substantially greater cumulative hazard relative to both the medium and high eCRF groups. Moreover, the log-rank test results (*p* < 0.0001 for HRS and ELSA, *p* = 0.0002 for CHARLS) indicated that the differences in cumulative hazard among the eCRF strata were statistically significant, further supporting the inverse association between higher eCRF and incident frailty (Figure 2).

As shown in Table 3, a consistent relationship was observed between baseline eCRF and incident frailty in all three study populations. The proportional hazards assumption for the key predictor, eCRF, was evaluated using Schoenfeld’s global test. No significant violations were detected in any of the cohorts (all *p*-values > 0.05; refer to Supplementary Tables S9–S11).

In the HRS cohort, following full covariate adjustment (Model 3), a per 1 SD increment in eCRF was associated with a 40% lower hazard of incident frailty (HR = 0.60; 95% CI, 0.54–0.68). When benchmarked against the low eCRF group, individuals classified with moderate eCRF displayed a 33% lower risk (HR = 0.67; 95% CI, 0.56–0.81), while those in the high eCRF stratum manifested a 50% risk decrease (HR = 0.50; 95% CI, 0.41–0.60).

Within the ELSA cohort, higher eCRF was significantly associated with a lower hazard of incident frailty. Per one-SD rise in eCRF, a 46% drop in risk was observed (HR = 0.54; 95% CI, 0.46–0.64). In comparison to the low eCRF reference category, participants with moderate eCRF had a 34% lower risk (HR = 0.66; 95% CI, 0.48–0.91), and those in the high eCRF category had a 70% lower risk (HR = 0.30; 95% CI, 0.21–0.43).

In the CHARLS cohort, after full adjustment for potential confounders (Model 3), each 1-SD increase in eCRF was associated with a 26% lower risk of incident frailty (HR = 0.74; 95% CI, 0.63–0.87). Compared with participants with low baseline eCRF, those with moderate eCRF had a 36% lower risk (HR = 0.64; 95% CI, 0.50–0.80), and participants in the high eCRF category had a 44% lower risk (HR = 0.56; 95% CI, 0.44–0.71). These findings indicate a graded inverse association between baseline eCRF and incident frailty across all three cohorts, with progressively lower hazards observed from the low to the moderate and high eCRF groups. The significant *p* for trend values in all three cohorts further support a dose-dependent pattern, suggesting that eCRF may have value for frailty risk stratification across diverse populations.

### 3.4. Dose–Response Relationship

**Figure 3:**
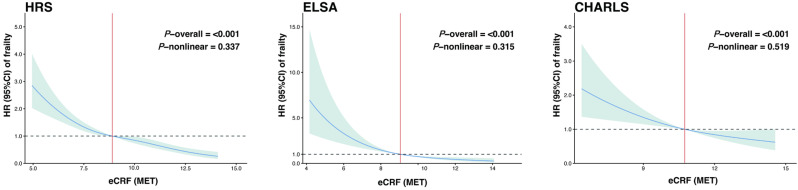
Restricted cubic spline curves depicting the association between eCRF and incident frailty. The blue solid line represents the estimated hazard ratio, the blue shaded area indicates the 95% confidence interval, the red vertical line marks the reference eCRF value at which HR = 1, and the horizontal dashed line indicates HR = 1. Abbreviations: HRS, Health and Retirement Study; ELSA, English Longitudinal Study of Ageing; CHARLS, China Health and Retirement Longitudinal Study; eCRF, estimated cardiorespiratory fitness; HR, hazard ratio; CI, confidence interval. The model adjusted for age, sex, education, employment status, marital status, co-residence with children, and alcohol consumption.

Figure 3 further demonstrated a graded inverse dose–response association between eCRF and incident frailty. Consistent with the categorical analyses, higher eCRF levels were associated with progressively lower hazards of incident frailty in all three cohorts after full adjustment (overall *p* < 0.001 for HRS, ELSA, and CHARLS). In addition, no significant nonlinearity was detected in any cohort (HRS: *p* = 0.337; ELSA: *p* = 0.315; CHARLS: *p* = 0.519), suggesting that the inverse association was broadly linear across the observed range of eCRF.

### 3.5. Subgroup Analyses

**Figure 4:**
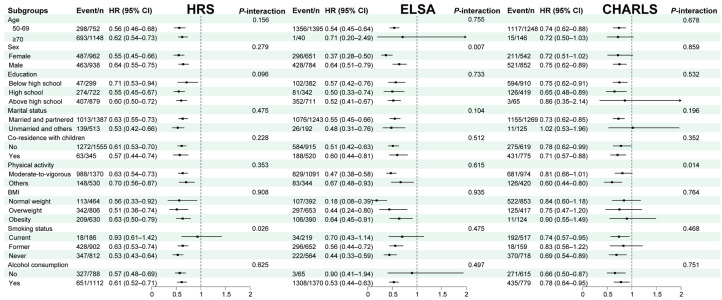
Subgroup and interaction analyses on the association of eCRF with the risk of incident frailty. Abbreviations: HRS, Health and Retirement Study; ELSA, English Longitudinal Study of Ageing; CHARLS, China Health and Retirement Longitudinal Study; eCRF, estimated cardiorespiratory fitness; BMI, body mass index; HR, hazard ratio; CI, confidence interval. The model adjusted for age, sex, education, employment status, marital status, co-residence with children, and alcohol consumption.

To examine effect modification, we performed stratified analyses to assess whether the association between eCRF, modeled as a continuous measure, and incident frailty varied across prespecified subgroups, as depicted in Figure 4.

In the HRS cohort, subgroup analyses indicated that the association was generally consistent across most categories. A statistically significant interaction was identified only for smoking history (interaction *p* = 0.026). More specifically, the inverse association between eCRF and incident frailty appeared more evident among never-smokers (HR = 0.53; 95% CI: 0.43–0.64) and former smokers (HR = 0.63; 95% CI: 0.53–0.74) than among current smokers.

Within the ELSA cohort, effect modification by sex was observed (interaction *p* = 0.007), with a stronger inverse association between eCRF and incident frailty among women (HR = 0.37; 95% CI, 0.28–0.50) per 1-SD higher eCRF.

In the CHARLS cohort, physical activity significantly modified the association between eCRF and incident frailty (interaction *p* = 0.014). Compared with participants engaging in moderate-to-vigorous physical activity, the inverse association between eCRF and incident frailty appeared stronger among those reporting lower physical activity levels; in this subgroup, each 1-SD increment in eCRF was associated with a lower risk of incident frailty (HR = 0.60; 95% CI, 0.44–0.80).

### 3.6. Sensitivity Analyses

Robustness of the main findings was further verified through a series of sensitivity analyses. First, after excluding individuals who developed frailty at the initial follow-up assessment, the association between eCRF and incident frailty remained consistent (Supplementary Table S12). Second, similar results were observed when participants with pre-existing cancer, lung, or CVD were excluded (Supplementary Table S13). Third, excluding individuals lacking important covariates at baseline did not substantially alter the observed associations (Supplementary Table S14). Fourth, consistent findings were obtained in analyses using multiple imputation methods (Supplementary Table S15). Finally, after additional adjustment for baseline FI as a continuous variable, the associations were attenuated but remained generally directionally consistent across the three cohorts (Supplementary Table S16).

## 4. Discussion

Using data from three major aging populations, namely the HRS, ELSA, and CHARLS, this investigation demonstrated a significant inverse relationship between elevated eCRF levels and frailty incidence. The persistence of this dose–response gradient across all studied populations highlights its robustness, and the relationship proved consistent across diverse demographic subgroups. Importantly, this association was not limited to a simple contrast between the highest and lowest eCRF groups; rather, we observed a graded inverse pattern across eCRF categories, with progressively lower frailty risk as eCRF increased. These findings suggest that higher eCRF may be relevant to frailty risk stratification and healthy aging in midlife and older age.

Our findings corroborate and broaden the existing evidence base. Previous studies have consistently identified CRF as a pivotal determinant of general health status, with well-documented inverse associations with all-cause mortality and cardiometabolic risk [30,31]. In line with this consensus, eCRF has been validated as a practical surrogate marker, particularly in reflecting metabolic health [32]. This analysis extends the observed inverse association of eCRF to the context of frailty, supporting the theoretical model that metabolic dysfunction contributes to the incidence of frailty [33,34,35]. Notably, recent investigations have directly linked cardiorespiratory health to the accumulation of deficits in frailty, offering dynamic insights into its role in incident frailty [20]. Collectively, this evidence suggests that higher eCRF may be associated with a lower risk of incident frailty, and more favorable metabolic health may be one possible explanation for this relationship. Importantly, the inverse association between eCRF and incident frailty remained directionally consistent after further adjustment for baseline FI, although the effect estimates were attenuated, suggesting that baseline deficit burden may partly, but not fully, explain the observed association. Moreover, our findings are also consistent with an inverse association between physical activity, a key component of the eCRF algorithm, and frailty, in line with recent studies [36,37]. Importantly, unlike most single-country studies, this is, to our knowledge, the first study to consistently demonstrate an inverse association between eCRF and incident frailty across aging populations from three continents, substantially enhancing the generalizability and robustness of the findings.

To explore potential mechanisms that may underlie the observed inverse association between eCRF and incident frailty, we considered several possible pathways. Higher eCRF may reflect better cardiorespiratory efficiency and oxygen delivery capacity, which in turn may help preserve physiological reserve and attenuate systemic inflammation [38]. At the organ and tissue level, higher eCRF may be associated with biological processes relevant to sarcopenia and muscle weakness, including the maintenance of muscle protein synthesis and muscle strength [39,40,41]. Behaviorally, higher eCRF may partly reflect greater engagement in moderate-to-vigorous physical activity, which has been associated with a lower risk of incident frailty [42]. Furthermore, exercise-induced enhancements in mitochondrial function and antioxidant defenses may decelerate cellular aging. In summary, eCRF may reflect multiple cardiorespiratory, muscular, metabolic, and cellular processes that could help explain its observed inverse association with frailty. The descriptive comparison of the individual eCRF components between frail and non-frail participants further supports this interpretation. In general, frail participants showed less favorable anthropometric, physiological, and behavioral profiles across cohorts, although some heterogeneity was observed for smoking, supporting the view that eCRF should be interpreted as an integrated composite marker rather than a proxy for any single component alone.

However, the association between eCRF and incident frailty was not uniform across subgroups. Subgroup analyses suggested some heterogeneity according to behavioral and social factors. Specifically, the inverse association between eCRF and incident frailty appeared stronger in certain subpopulations, including women [43], never smokers, and former smokers [44]. Of particular note, the inverse association between eCRF and incident frailty appeared strongest among individuals with low physical activity levels [36,37]. Nevertheless, these subgroup findings should be interpreted cautiously, because some stratification variables, particularly physical activity and smoking, are also components of the eCRF algorithm itself, which complicates interpretation and raises the possibility of conceptual overlap. Therefore, these findings should be regarded as exploratory and hypothesis-generating rather than as definitive evidence of differential associations across subgroups.

The present study has several clinical and public health implications. As a non-invasive and practical metric derived from readily available parameters such as age, BMI, and physical activity, eCRF may have value for integration into routine geriatric assessment and cardiovascular risk management frameworks [15]. Clinicians may use eCRF to help identify individuals at elevated risk at an early stage and to inform more individualized assessment and counseling. In addition, the observed association between eCRF and incident frailty suggests that further investigation into modifiable frailty-related pathways is warranted. At the population level, the clear dose–response relationship between eCRF and incident frailty further supports the relevance of physical activity-related and fitness-related factors in frailty risk assessment. These findings provide a rationale for further evaluating whether strategies aimed at improving CRF-related factors may contribute to healthy aging and reduce frailty burden [45]. Future studies should further examine whether interventions targeting modifiable components related to eCRF can influence frailty trajectories and management outcomes.

Several limitations should be acknowledged. First, the substantial exclusion of participants due to missing data, particularly for variables required for eCRF and FI derivation, may have introduced selection bias. Although the multiple imputation analyses yielded generally consistent findings, the main analyses were based on complete-case data; therefore, residual bias related to missingness cannot be fully excluded. Second, because frailty was assessed at discrete follow-up waves, the exact time of incident frailty was not directly observed. We therefore approximated event time using the first wave at which frailty was identified and applied standard Cox regression; however, interval censoring was not explicitly modeled and may have introduced some imprecision in the time-to-event estimates. Third, as an estimated measure, eCRF is susceptible to non-differential misclassification; however, given its validated algorithm, such errors are likely to be random and are not expected to have caused substantial bias. Fourth, although the observational design precludes definitive causal inference, we strengthened the robustness of the findings through repeated follow-ups, extensive confounding adjustment, and multiple sensitivity analyses. Fifth, certain eCRF parameters were based on self-report, which may have introduced recall bias, although standardized instruments were used to minimize this concern. In addition, subgroup analyses stratified by variables such as physical activity and smoking should be interpreted cautiously, because these variables are also embedded in the eCRF algorithm and may introduce conceptual overlap in the interpretation of effect heterogeneity. Additionally, while the FI used here may differ in specific item content from other indices [46,47], their theoretical foundations are consistent, supporting comparability. Finally, although the cohorts included major Eastern and Western aging populations, further validation is still needed in other healthcare systems and cultural settings.

This study has several important strengths. We used large, nationally representative longitudinal cohorts from the United States, England, and China, which enhanced the robustness, temporal inference, and cross-population relevance of the findings. The consistency of the observed inverse association across these diverse settings further supports the external validity of the results. In addition, the use of a standardized Frailty Index enhanced the comparability of frailty assessment across cohorts.

## 5. Conclusions

Higher eCRF was associated with a lower risk of incident frailty among middle-aged and older adults across three nationally representative cohorts. As an accessible and non-invasive metric based on routinely available parameters, eCRF may contribute to practical frailty risk stratification in clinical and public health settings.

## Figures and Tables

**Table 1:** Baseline characteristics of participants included in the longitudinal analysis of incident frailty in HRS, ELSA, and CHARLS.

Characteristics	HRS	ELSA	CHARLS	*p*-Value
Number (%)	1900	4816	1436	
eCRF, mean (SD)	9.33 (2.10)	9.19 (1.85)	10.78 (1.94)	<0.001
Age, mean (SD), years	71.54 (4.87)	63.87 (7.83)	60.28 (6.88)	<0.001
**Sex, *n* (%)**				<0.001
Male	938 (49.37%)	2347 (48.73%)	885 (61.63%)	
Female	962 (50.63%)	2469 (51.27%)	551 (38.37%)	
**Education, *n* (%)**				<0.001
Below high school	299 (15.74%)	1669 (37.91%)	939 (65.39%)	
High school	722 (38.00%)	946 (21.49%)	429 (29.87%)	
Above high school	879 (46.26%)	1787 (40.60%)	68 (4.74%)	
**Employment, *n* (%)**				<0.001
Unemployed	132 (6.95%)	42 (2.15%)	19 (1.33%)	
Working or retired	1768 (93.05%)	1908 (97.85%)	1410 (98.67%)	
**Marital status, *n* (%)**				<0.001
Unmarried and others	513 (27.00%)	1171 (24.32%)	142 (9.89%)	
Married and partnered	1387 (73.00%)	3644 (75.68%)	1294 (90.11%)	
**Co-residence with children, *n* (%)**				<0.001
No	1555 (81.84%)	3196 (77.20%)	622 (44.37%)	
Yes	345 (18.16%)	944 (22.80%)	780 (55.63%)	
**Smoking status, *n* (%)**				<0.001
Never	812 (42.74%)	1879 (39.02%)	733 (51.04%)	
Former	902 (47.47%)	2310 (47.97%)	162 (11.28%)	
Current	186 (9.79%)	627 (13.02%)	541 (37.67%)	
**Alcohol consumption, *n* (%)**				<0.001
No	788 (41.47%)	316 (7.06%)	627 (43.69%)	
Yes	1112 (58.53%)	4162 (92.94%)	808 (56.31%)	
**Physical activity, *n* (%)**				<0.001
Others	530 (27.89%)	1226 (25.46%)	434 (30.22%)	
Moderate-to-vigorous	1370 (72.11%)	3590 (74.54%)	1002 (69.78%)	
BMI, mean (SD), kg/m^2^	28.37 (4.76)	27.66 (4.48)	23.54 (3.41)	<0.001
WC, mean (SD), cm	99.39 (13.03)	95.05 (12.52)	84.53 (12.06)	<0.001
rHR, mean (SD), bpm	69.22 (10.70)	66.87 (10.65)	71.60 (10.23)	<0.001
Cancer, *n* (%)	273 (14.37%)	288 (5.98%)	11 (0.77%)	<0.001
Lung disease, *n* (%)	99 (5.21%)	171 (3.55%)	103 (7.17%)	<0.001
CVD, *n* (%)	411 (21.63%)	634 (13.16%)	140 (9.75%)	<0.001
FI, median (IQR)	0.12 (0.08, 0.18)	0.08 (0.04, 0.14)	0.13 (0.08, 0.18)	<0.001
Follow-up time, mean (SD), years	8.14 (4.18)	9.57 (4.62)	4.90 (2.03)	<0.001

Abbreviations: SD: standard deviation, HRS: Health and Retirement Study, ELSA: English Longitudinal Study of Ageing, CHARLS: China Health and Retirement Longitudinal Study, eCRF: estimated cardiorespiratory fitness, BMI: body mass index, WC: waist circumference, rHR: resting heart rate, FI: frailty index. Bold text is used to indicate subgroup categories for clarity.

**Table 2:** Cross-sectional associations of eCRF with frailty at baseline.

	Event	Total	Event/Total%	OR	95% CI	*p*-Value
HRS						
Low eCRF	230	521	44.15	1	Ref	
Medium eCRF	238	1039	22.91	0.40	0.32–0.50	<0.001
High eCRF	187	1133	16.50	0.30	0.23–0.38	<0.001
ELSA						
Low eCRF	428	1265	33.83	1	Ref	
Medium eCRF	439	2572	17.07	0.42	0.25–0.73	0.002
High eCRF	189	2542	7.44	0.11	0.05–0.24	<0.001
CHARLS						
Low eCRF	176	509	34.58	1	Ref	
Medium eCRF	256	1008	25.40	0.61	0.48–0.79	<0.001
High eCRF	202	849	23.79	0.56	0.43–0.72	<0.001

Abbreviations: HRS: Health and Retirement Study, ELSA: English Longitudinal Study of Ageing, CHARLS: China Health and Retirement Longitudinal Study, eCRF: estimated cardiorespiratory fitness, OR: Odds Ratio, CI: Confidence Interval. Fully adjusted for age, sex, education, employment status, marital status, co-residence with children, and alcohol consumption.

**Table 3:** Associations of eCRF with incident frailty.

	Events/*n*	Model 1		Model 2		Model 3	
		HR (95% CI)	*p*-Value	HR (95% CI)	*p*-Value	HR (95% CI)	*p*-Value
HRS							
eCRF per 1-SD	871/1900	0.79 (0.74–0.85)	<0.001	0.58 (0.52–0.65)	<0.001	0.60 (0.54–0.68)	<0.001
Low eCRF level	175/272	reference		reference		reference	
Moderate eCRF level	360/748	0.68 (0.56–0.81)	<0.001	0.66 (0.55–0.79)	<0.001	0.67 (0.56–0.81)	<0.001
High eCRF level	336/880	0.48 (0.40–0.58)	<0.001	0.47 (0.39–0.56)	<0.001	0.50 (0.41–0.60)	<0.001
*p* for trend			<0.001		<0.001		<0.001
ELSA							
eCRF per 1-SD	1328/4816	0.53 (0.50–0.56)	<0.001	0.56 (0.52–0.61)	<0.001	0.54 (0.46–0.64)	<0.001
Low eCRF level	299/734	reference		reference		reference	
Moderate eCRF level	580/1920	0.66 (0.58–0.76)	<0.001	0.60 (0.52–0.69)	<0.001	0.66 (0.48–0.91)	0.010
High eCRF level	449/2162	0.41 (0.35–0.48)	<0.001	0.37 (0.32–0.42)	<0.001	0.30 (0.21–0.43)	<0.001
*p* for trend			<0.001		<0.001		<0.001
CHARLS							
eCRF per 1-SD	489/1436	0.72 (0.66–0.79)	<0.001	0.75 (0.64–0.88)	<0.001	0.74 (0.63–0.87)	<0.001
Low eCRF level	113/266	reference		reference		reference	
Moderate eCRF level	210/625	0.70 (0.56–0.88)	0.002	0.66 (0.52–0.83)	<0.001	0.64 (0.50–0.80)	<0.001
High eCRF level	216/545	0.61 (0.48–0.77)	<0.001	0.59 (0.46–0.75)	<0.001	0.56 (0.44–0.71)	<0.001
*p* for trend			<0.001		<0.001		<0.001

Abbreviations: HRS: Health and Retirement Study, ELSA: English Longitudinal Study of Ageing, CHARLS: China Health and Retirement Longitudinal Study, eCRF: estimated cardiorespiratory fitness, HR: hazard ratio, CI: confidence interval. Model 1: unadjusted. Model 2: adjusted for age and sex. Model 3: fully adjusted for age, sex, education, employment status, marital status, co-residence with children, and alcohol consumption.

## Data Availability

The data that support the findings of this study are openly available in the following repositories: HRS (https://hrs.isr.umich.edu), ELSA (www.elsa-project.ac.uk), and CHARLS (http://charls.pku.edu.cn/en).

## Supplementary Materials

The following supporting information can be downloaded at: https://www.mdpi.com/article/10.3390/healthcare14091169/s1, Figure S1: The distribution of eCRF in HRS, ELSA, and CHARLS; Table S1: The 30 items used to construct the frailty index; Table S2: The missing number and rate of covariates for baseline analyses; Table S3: Baseline characteristics of participants included in the cross-sectional study in HRS, ELSA, and CHARLS; Table S4: Characteristics of included and excluded participants for missing data and frailty status at baseline; Table S5: Comparison of eCRF components between frail and non-frail participants at baseline in HRS, ELSA, and CHARLS; Tables S6–S8: Baseline characteristics of HRS, ELSA, and CHARLS participants stratified by eCRF groups based on quintiles; Tables S9–S11: Schoenfeld residuals test for the Cox proportional hazards assumption in HRS, ELSA, and CHARLS; Table S12: Association of eCRF with risks of incident frailty after excluding participants with incident frailty during the first wave of follow-up; Table S13: Association of eCRF with risks of incident frailty after excluding participants with cancer, lung diseases or cardiovascular disease at baseline; Table S14: Association of eCRF with risks of incident frailty after excluding participants missing important covariates at baseline; Table S15: Association of eCRF with risks of incident frailty with multiple imputation; Table S16: Association of eCRF with risks of incident frailty after additional adjustment for baseline FI.

## Abbreviations

The following abbreviations are used in this manuscript:

eCRF	estimated cardiorespiratory fitness
HRS	Health and Retirement Study
ELSA	English Longitudinal Study of Ageing
CHARLS	China Health and Retirement Longitudinal Study
FI	Frailty Index
BMI	body mass index
WC	waist circumference
rHR	resting heart rate
CVD	cardiovascular disease
