# Supplementary material for: Estimated Cardiorespiratory Fitness and Risk of Incident Frailty in Middle-Aged and Older Adults: A Cross-National Longitudinal Cohort Study
Source: Healthcare (Basel). 2026 Apr 27;14(9):1169. doi: 10.3390/healthcare14091169 (PMC13163999; doi:10.3390/healthcare14091169)
Supplement: Supplementary file 1 [file healthcare-14-01169-s001.zip › Supplementary_Material.pdf]

Supplementary Material

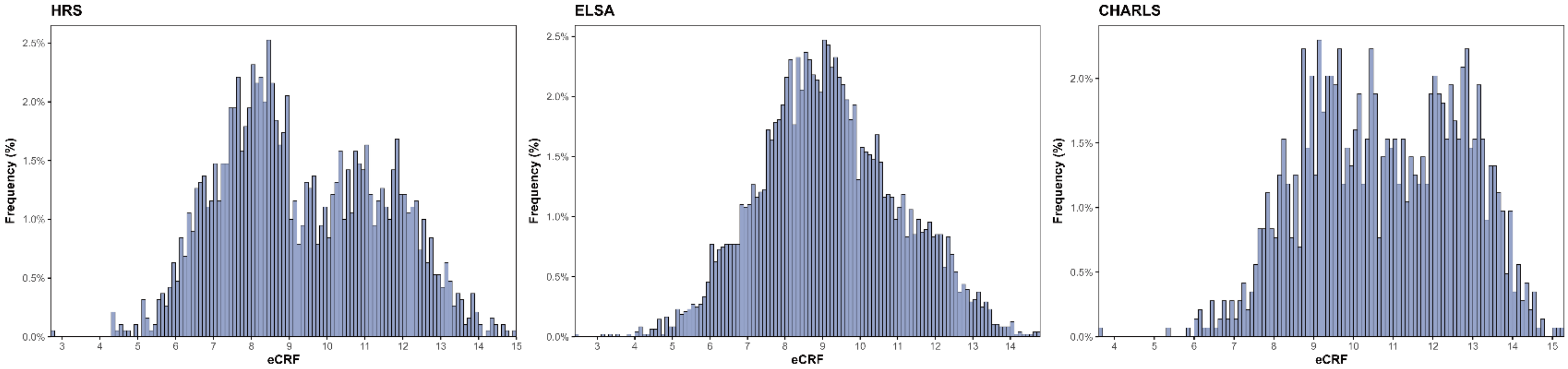

Figure S1. The distribution of eCRF in HRS, ELSA, and CHARLS. Abbreviations: HRS, Health and Retirement Study; ELSA, English Longitudinal Study of Ageing; CHARLS, China Health and Retirement Longitudinal Study; eCRF, estimated cardiorespiratory fitness.

Supplemental Methods. Detailed information of three cohorts.

Study designs of the HRS, ELSA, and CHARLS

The Health and Retirement Study (HRS) is a large, nationally representative prospective cohort study in the United States. Launched in 1992, it initially enrolled middle-aged and older adults aged 51 to 61. In 1993, the HRS integrated the Study of Assets and Health Dynamics Among the Oldest Old (AHEAD), which focused on adults aged 70 years and older. In 1998, the study further merged with the Children of the Great Depression and War Babies cohorts, resulting in a sample fully representative of U.S. adults aged 50 and above. Since its inception, the HRS has conducted follow-up surveys every two years to systematically collect data on sociodemographic characteristics, lifestyle, and health status. Prior to 2004 (i.e., Wave 7), most data were collected via telephone, although face-to-face interviews were available for participants aged 80 and above. Starting in 2006 (Wave 8), the survey mode was adjusted: half of the participants underwent an enhanced face-to-face interview (EFTF), while the other half continued to be interviewed by telephone. During the EFTF, trained staff performed a series of physical measurements—including height, weight, waist circumference, blood pressure, and grip strength—and collected blood samples for the analysis of five biomarkers: total cholesterol, high-density lipoprotein (HDL) cholesterol, glycosylated hemoglobin (HbA1c), C-reactive protein, and cystatin C. The study has maintained biennial follow-ups since its first wave, with data collection primarily conducted by telephone in the initial phases.

The English Longitudinal Study of Ageing (ELSA) is a prospective cohort study conducted in the United Kingdom. The baseline survey, completed in 2002–2003, included a nationally representative sample of 11,391 individuals aged 50 years and older. Detailed information on sociodemographic background, lifestyle, and health status was collected through a combination of face-to-face interviews and self-administered questionnaires administered by trained interviewers. Starting from the

second wave (2004–2005), the study introduced nurse home visits. Physical measurements were obtained from a core sample of 7,666 participants, including anthropometric data (e.g., height, weight, waist-to-hip ratio), blood pressure, grip strength, and blood samples for assessing biomarkers such as total cholesterol, HDL and LDL cholesterol, glycosylated hemoglobin, fasting blood glucose, and C-reactive protein. Follow-up surveys have been conducted every two years, with nurse visits repeated every four years since the second wave, establishing a systematic long-term follow-up mechanism.

The China Health and Retirement Longitudinal Study (CHARLS) is a national prospective cohort survey that conducted its first wave of data collection in 2011. Using a multi-stage probability sampling strategy, the study covered 28 provincial-level administrative regions in China and enrolled 17,708 nationally representative participants. The target population consisted of adults aged 45 years and older, though some respondents aged 40 to 44 were also included at baseline. All participants were interviewed at home by uniformly trained enumerators using a standardized questionnaire to gather information on sociodemographic characteristics, lifestyle, and health status. Among the total sample, 13,978 individuals completed physical measurements—such as height, weight, waist and hip circumference, blood pressure, and grip strength—and 11,847 provided blood samples for laboratory tests of biomarkers including total cholesterol, HDL and LDL cholesterol, glycosylated hemoglobin, fasting blood glucose, and C-reactive protein. Follow-up surveys were conducted in 2013 (Wave 2), 2015 (Wave 3), and 2018 (Wave 4), with repeated questionnaires and physical measurements in each wave. Blood samples were collected again during the 2015 follow-up for corresponding biomarker analysis.

Descriptions of covariates

For the purpose of this study, marital status was classified into two categories: married or in a partnership, and others (which included separated, divorced, widowed, or unmarried individuals). Educational attainment was categorized into three levels: did

not complete high school, high school graduate, and college degree or higher. Specifically, in the HRS, the post-secondary or higher group comprised respondents who had earned a high school diploma or General Educational Development (GED) certificate and had completed more than 12 years of education. In the ELSA, this category included individuals who had attended college or attained a college degree or higher. In the CHARLS, it encompassed participants with a two- or three-year college degree, a four-year undergraduate degree, or a master's or doctoral degree.

Smoking behavior was classified into three categories: current smoker, former smoker, and never smoker. During data collection, participants were asked whether they had ever smoked cigarettes, used a pipe, or chewed tobacco. Smoking was explicitly defined as having smoked more than 100 cigarettes in one's lifetime. Respondents who answered "no" were classified as never smokers. Those who answered "yes" were further asked whether they currently maintained the habit or had quit. Individuals who reported having quit were categorized as former smokers, while those who indicated they were still smoking were classified as current smokers.

Regarding alcohol consumption, respondents were asked whether they had ever consumed any alcoholic beverages. Those who reported never having consumed alcohol were categorized as non-drinkers, while those who had consumed alcohol were classified as drinkers.

Physical activity levels were divided into active and inactive groups. Across all three surveys, participants were asked whether they engaged in vigorous, moderate, or light physical activity lasting at least 10 minutes, at least once per week. The active group included those who reported engaging in vigorous- or moderate-intensity physical activity more than once per week, while all others were categorized as inactive.

Table S1. The 30 items used to construct the frailty index.

<table>
<tr><td rowspan="2">No</td><td colspan="3">Description of the item</td><td rowspan="2">Cut-off value</td></tr>
<tr><td>CHARLS</td><td>ELSA</td><td>HRS</td></tr>
<tr><td>1</td><td colspan="3">Self-reported physician diagnosed hypertension</td><td>Yes = 1, No = 0</td></tr>
<tr><td>2</td><td colspan="3">Self-reported physician diagnosed diabetes</td><td>Yes = 1, No = 0</td></tr>
<tr><td>3</td><td colspan="3">Self-reported physician diagnosed heart disease</td><td>Yes = 1, No = 0</td></tr>
<tr><td>4</td><td colspan="3">Self-reported physician diagnosed stroke</td><td>Yes = 1, No = 0</td></tr>
<tr><td>5</td><td colspan="3">Self-reported physician diagnosed cancer</td><td>Yes = 1, No = 0</td></tr>
<tr><td>6</td><td colspan="3">Self-reported physician diagnosed arthritis</td><td>Yes = 1, No = 0</td></tr>
<tr><td>7</td><td colspan="3">Self-reported physician diagnosed chronic lung disease</td><td>Yes = 1, No = 0</td></tr>
<tr><td>8</td><td colspan="3">Self-reported physician diagnosed any emotional, nervous, or psychiatric problems</td><td>Yes = 1, No = 0</td></tr>
<tr><td>9</td><td colspan="3">Self-reported physician diagnosed memory-related disease, including Alzheimer’s disease or dementia, organic brain senility, or other serious memory impairment</td><td>Yes = 1, No = 0</td></tr>
<tr><td>10</td><td>Self-reported vision problems</td><td colspan="2">Self-reported eyesight (while using lenses if appropriate)</td><td>Yes = 1, No = 0 in the CHARLS; Poor or fair = 1, excellent, very good, or good = 0 in the ELSA and HRS</td></tr>
<tr><td>11</td><td>Self-reported hearing problems</td><td colspan="2">Self-reported hearing (while using hearing aid
if appropriate)</td><td>Yes = 1, No = 0 in the CHARLS; Poor or fair = 1, excellent, very good, or good = 0 in the ELSA and HRS</td></tr>
<tr><td>12</td><td colspan="3">Self-reported general health status</td><td>Very poor or poor = 1,
Very good, good, or fair = 0</td></tr>
<tr><td>13</td><td colspan="3">Difficulty with dressing</td><td>Yes = 1, No = 0</td></tr>
<tr><td>14</td><td colspan="3">Difficulty with bathing or showering</td><td>Yes = 1, No = 0</td></tr>
<tr><td>15</td><td colspan="3">Difficulty with eating</td><td>Yes = 1, No = 0</td></tr>
<tr><td>16</td><td colspan="3">Difficulty with getting in and out of bed</td><td>Yes = 1, No = 0</td></tr>
</table>

<table>
<tr><td>17</td><td colspan="2">Difficulty with using the toilet</td><td>Yes = 1, No = 0</td></tr>
<tr><td>18</td><td colspan="2">Difficulty with managing money</td><td>Yes = 1, No = 0</td></tr>
<tr><td>19</td><td colspan="2">Difficulty with taking medication</td><td>Yes = 1, No = 0</td></tr>
<tr><td>20</td><td colspan="2">Difficulty with shopping for groceries</td><td>Yes = 1, No = 0</td></tr>
<tr><td>21</td><td colspan="2">Difficulty with preparing meals</td><td>Yes = 1, No = 0</td></tr>
<tr><td>22</td><td colspan="2">Mobility: difficulty with walking 100 yards or one block</td><td>Yes = 1, No = 0</td></tr>
<tr><td>23</td><td colspan="2">Mobility: difficulty with getting up from a chair after sitting for long periods</td><td>Yes = 1, No = 0</td></tr>
<tr><td>24</td><td colspan="2">Mobility: difficulty with climbing several flights of stairs without resting</td><td>Yes = 1, No = 0</td></tr>
<tr><td>25</td><td colspan="2">Mobility: difficulty with lifting or carrying weights over 10 pounds</td><td>Yes = 1, No = 0</td></tr>
<tr><td>26</td><td colspan="2">Mobility: difficulty with picking up a coin from the table</td><td>Yes = 1, No = 0</td></tr>
<tr><td>27</td><td colspan="2">Mobility: difficulty with stooping, kneeling, or crouching</td><td>Yes = 1, No = 0</td></tr>
<tr><td>28</td><td colspan="2">Mobility: difficulty with reaching arms above shoulder level</td><td>Yes = 1, No = 0</td></tr>
<tr><td>29</td><td>Depression: CESD-10 questionnaire</td><td>Depression: CESD-8 questionnaire</td><td>CESD-10 >10 = 1, ≤10 = 0 in the CHARLS; CESD-8 ≥ 4 = 1, <4 = 0 in the ELSA and HRS</td></tr>
<tr><td>30</td><td colspan="2">Cognition: (total score- (immediate and delayed word recall + date naming)) / total score</td><td>Continuous variable, ranging from 0 to 1</td></tr>
</table>

Depression was assessed by the Center for Epidemiologic Studies Depression Scale (CESD). In the CHARLS, CESD-10 was used, and the total score ranged from 0 to 30. In the ELSA and HRS, CESD-8 was used, and the total score ranged from 0 to 8. A higher score indicated more severe depressive symptoms.

The cognitive assessment comprised three components: immediate word recall, delayed word recall, and temporal orientation. In the word recall tasks, participants were required to memorize a list of ten words (used across HRS, CHARLS, and ELSA studies). The temporal orientation section evaluated the ability

to accurately report the current date—including day of the week, month, and year—as implemented in the HRS, CHARLS, and ELSA surveys. Each correct response was assigned one point. Cognitive performance was quantified using the formula: (Total score − (Immediate Word Recall + Delayed Word Recall + Date Naming)) / Total score, where the total score represents the theoretical maximum achievable across all sections. Higher values on this metric indicate a lower level of cognitive functioning.

HRS, Health and Retirement Study; ELSA, English Longitudinal Study of Ageing; CHARLS, China Health and Retirement Longitudinal Study.

Table S2. The missing number and rate of covariates for baseline analyses.

**Variables**	**HRS (n=1,900)**	**ELSA (n=4,816)**	**CHARLS (n=1,436)**
Age	0(0.00%)	0(0.00%)	0(0.00%)
Sex	0(0.00%)	0(0.00%)	0(0.00%)
Education	0(0.00%)	414(8.60%)	0(0.00%)
Employment	0(0.00%)	2866(59.51%)	7(0.49%)
Marital status	0(0.00%)	1(0.02%)	0(0.00%)
Co-residence with children	0(0.00%)	676(14.04%)	34(2.37%)
Smoking status	0(0.00%)	0(0.00%)	0(0.00%)
Alcohol consumption	0(0.00%)	338(7.02%)	1(0.07%)
Physical activity	0(0.00%)	0(0.00%)	0(0.00%)
BMI	0(0.00%)	0(0.00%)	0(0.00%)
WC	0(0.00%)	0(0.00%)	0(0.00%)
rHR	0(0.00%)	0(0.00%)	0(0.00%)

Cancer	0(0.00%)	0(0.00%)	0(0.00%)
Lung disease	0(0.00%)	0(0.00%)	0(0.00%)
CVD	0(0.00%)	0(0.00%)	0(0.00%)

Abbreviations: HRS, Health and Retirement Study; ELSA, English Longitudinal Study of Ageing; CHARLS, China Health and Retirement Longitudinal Study; BMI, body mass index; WC, waist circumference; rHR, resting heart rate; CVD, cardiovascular disease.

Table S3. Baseline characteristics of participants included in the cross-sectional study in HRS, ELSA, and CHARLS.

**Characteristics**	**HRS**	**ELSA**	**CHARLS**	***P*-value**
**Number (%)**	2693	6379	2366	
**eCRF, mean (SD)**	9.11 (2.17)	8.92 (1.91)	10.49 (2.01)	<0.001
**Age, mean (SD), years**	71.92 (5.16)	64.54 (8.04)	61.12 (7.23)	<0.001
**Sex, *n* (%)**				<0.001
Male	1333 (49.50%)	3065 (48.05%)	1360 (57.48%)	
Female	1360 (50.50%)	3314 (51.95%)	1006 (42.52%)	
**Education, *n* (%)**				<0.001
Below high school	542 (20.13%)	2537 (43.58%)	1627 (68.77%)	
High school	1034 (38.40%)	1178 (20.23%)	643 (27.18%)	
Above high school	1117 (41.48%)	2107 (36.19%)	96 (4.06%)	
**Employment, *n* (%)**				<0.001
Unemployed	215 (7.98%)	49 (2.20%)	36 (1.54%)	
Working or retired	2478 (92.02%)	2175 (97.80%)	2309 (98.46%)	
**Marital status, *n* (%)**				<0.001
Unmarried and others	824 (30.60%)	1668 (26.15%)	294 (12.43%)	
Married and partnered	1869 (69.40%)	4710 (73.85%)	2072 (87.57%)	
**Co-residence with children, *n* (%)**				<0.001

Characteristics	HRS	ELSA	CHARLS	*P*-value
No	2138 (79.39%)	4320 (78.46%)	1090 (47.12%)	
Yes	555 (20.61%)	1186 (21.54%)	1223 (52.88%)	
**Smoking status, *n* (%)**				<0.001
Never	1098 (40.77%)	2339 (36.67%)	1262 (53.34%)	
Former	1309 (48.61%)	3093 (48.49%)	270 (11.41%)	
Current	286 (10.62%)	946 (14.83%)	834 (35.25%)	
**Alcohol consumption, *n* (%)**				<0.001
No	1264 (46.94%)	551 (9.45%)	1054 (44.57%)	
Yes	1429 (53.06%)	5280 (90.55%)	1311 (55.43%)	
**Physical activity, *n* (%)**				0.021
Others	958 (35.57%)	2082 (32.64%)	810 (34.23%)	
Moderate-to-vigorous	1735 (64.43%)	4297 (67.36%)	1556 (65.77%)	
**BMI, mean (SD), kg/m²**	28.85 (5.23)	28.06 (4.73)	23.70 (3.43)	<0.001
**WC, mean (SD), cm**	39.72 (5.42)	96.09 (12.90)	85.27 (12.45)	<0.001
**rHR, mean (SD), bpm**	69.57 (11.30)	67.54 (11.03)	71.96 (10.64)	<0.001
**Cancer, *n* (%)**	458 (17.01%)	455 (7.13%)	27 (1.14%)	<0.001
**Lung disease, *n* (%)**	251 (9.32%)	376 (5.89%)	248 (10.48%)	<0.001
**CVD, *n* (%)**	808 (30.00%)	1150 (18.03%)	358 (15.13%)	<0.001

Characteristics	HRS	ELSA	CHARLS	*P*-value
**FI, median (IQR)**	0.15 (0.08, 0.25)	0.10 (0.05, 0.18)	0.16 (0.11, 0.25)	<0.001
**Follow-up time, mean (SD), years**	7.02 (4.38)	8.54 (4.96)	4.14 (1.62)	<0.001

Abbreviations: SD standard deviation, HRS Health and Retirement Study, ELSA English Longitudinal Study of Ageing, CHARLS China Health and Retirement Longitudinal Study, eCRF estimated cardiorespiratory fitness, BMI body mass index, WC waist circumference, rHR resting heart rate, FI frailty index.

Bold text is used to indicate subgroup categories for clarity.

Table S4. Characteristics of included and excluded participants for missing data and frailty status at baseline.

Characteristics	HRS		*P*-value	ELSA		*P*-value	CHARLS		*P*-value
	Include	Exclude		Include	Exclude		Include	Exclude	
Number (%)	1900	31779		4816	4355		1436	12030	
**eCRF, mean (SD)**	9.33 (2.10)	9.09 (2.61)	<0.001	9.19 (1.85)	7.52 (2.10)	<0.001	10.78 (1.94)	9.91 (2.12)	<0.001
**Age, mean (SD), years**	71.54 (4.87)	71.24 (13.88)	0.029	63.87 (7.83)	68.96 (11.30)	<0.001	60.28 (6.88)	62.42 (8.70)	<0.001
**Sex, *n* (%)**			<0.001			<0.001			<0.001
Male	938 (49.37%)	14075 (44.29%)		2347 (48.73%)	1737 (39.89%)		885 (61.63%)	5811 (48.30%)	
Female	962 (50.63%)	17704 (55.71%)		2469 (51.27%)	2618 (60.11%)		551 (38.37%)	6219 (51.70%)	
**Education, *n* (%)**			<0.001			<0.001			<0.001
Below high school	299 (15.74%)	9114 (28.70%)		1669 (37.91%)	2324 (58.51%)		939 (65.39%)	8775 (72.98%)	
High school	722 (38.00%)	10556 (33.24%)		946 (21.49%)	619 (15.58%)		429 (29.87%)	2726 (22.67%)	
Above high school	879 (46.26%)	12091 (38.07%)		1787 (40.60%)	1029 (25.91%)		68 (4.74%)	523 (4.35%)	
**Employment, *n* (%)**			<0.001			0.335			0.005
Unemployed	132 (6.95%)	2122 (13.19%)		42 (2.15%)	27 (2.81%)		19 (1.33%)	303 (2.59%)	

Working or retired	1768 (93.05%)	13969 (86.81%)		1908 (97.85%)	934 (97.19%)		1410 (98.67%)	11399 (97.41%)	
**Marital status, *n* (%)**			<0.001			<0.001			<0.001
Unmarried and others	513 (27.00%)	5859 (36.42%)		1171 (24.32%)	1664 (38.21%)		142 (9.89%)	1912 (15.91%)	
Married and partnered	1387 (73.00%)	10230 (63.58%)		3644 (75.68%)	2691 (61.79%)		1294 (90.11%)	10104 (84.09%)	
**Co-residence with children, *n* (%)**			<0.001			0.002			0.887
No	1555 (81.84%)	11183 (69.50%)		3196 (77.20%)	2955 (80.17%)		622 (44.37%)	5217 (44.60%)	
Yes	345 (18.16%)	4908 (30.50%)		944 (22.80%)	731 (19.83%)		780 (55.63%)	6479 (55.40%)	
**Smoking status, *n* (%)**			<0.001			<0.001			<0.001
Never	812 (42.74%)	6845 (42.94%)		1879 (39.02%)	1481 (34.03%)		733 (51.04%)	7078 (61.77%)	
Former	902 (47.47%)	6907 (43.33%)		2310 (47.97%)	2087 (47.95%)		162 (11.28%)	1056 (9.22%)	
Current	186 (9.79%)	2188 (13.73%)		627 (13.02%)	784 (18.01%)		541 (37.67%)	3325 (29.02%)	
**Alcohol consumption, *n* (%)**			<0.001			<0.001			<0.001
No	788 (41.47%)	8327 (51.77%)		316 (7.06%)	545 (15.83%)		627 (43.69%)	7392 (62.04%)	
Yes	1112 (58.53%)	7758 (48.23%)		4162 (92.94%)	2898 (84.17%)		808 (56.31%)	4522 (37.96%)	

**Physical activity, *n* (%)**			<0.001			<0.001			<0.001
Others	530 (27.89%)	7220 (44.93%)		1226 (25.46%)	2223 (52.57%)		434 (30.22%)	1606 (41.48%)	
Moderate-to-vigorous	1370 (72.11%)	8851 (55.07%)		3590 (74.54%)	2006 (47.43%)		1002 (69.78%)	2266 (58.52%)	
**BMI, mean (SD), kg/m²**	28.37 (4.76)	29.26 (6.04)	<0.001	27.66 (4.48)	28.47 (5.59)	<0.001	23.54 (3.41)	23.66 (21.10)	0.606
**WC, mean (SD), cm**	39.13 (5.13)	39.85 (6.31)	<0.001	95.05 (12.52)	96.82 (14.23)	<0.001	84.53 (12.06)	84.30 (12.73)	0.502
**rHR, mean (SD), bpm**	69.22 (10.70)	70.97 (11.72)	<0.001	66.87 (10.65)	70.11 (12.06)	<0.001	71.60 (10.23)	72.29 (10.69)	0.017
**Cancer, *n* (%)**	273 (14.37%)	2271 (14.11%)	0.790	288 (5.98%)	381 (8.75%)	<0.001	11 (0.77%)	122 (1.03%)	0.424
**Lung disease, *n* (%)**	99 (5.21%)	1512 (9.40%)	<0.001	171 (3.55%)	410 (9.42%)	<0.001	103 (7.17%)	1360 (11.44%)	<0.001
**CVD, *n* (%)**	411 (21.63%)	4790 (29.77%)	<0.001	634 (13.16%)	1303 (29.93%)	<0.001	140 (9.75%)	2015 (17.00%)	<0.001
**FI, median (IQR)**	0.12 (0.08, 0.18)	0.21 (0.11, 0.32)	<0.001	0.08 (0.04, 0.14)	0.22 (0.08, 0.35)	<0.001	0.13 (0.08, 0.18)	0.18 (0.11, 0.28)	<0.001
**Follow-up time, mean (SD), years**	8.14 (4.18)	7.89 (4.57)	0.013	9.57 (4.62)	5.20 (4.36)	<0.001	4.90 (2.03)	4.33 (2.08)	<0.001

Abbreviations: SD standard deviation, HRS Health and Retirement Study, ELSA English Longitudinal Study of Ageing, CHARLS China Health and Retirement Longitudinal Study, eCRF estimated cardiorespiratory fitness, BMI body mass index, WC waist circumference, rHR resting heart rate, FI frailty index.

Bold text is used to indicate subgroup categories for clarity.

Table S5. Comparison of eCRF components between frail and non-frail participants at baseline in HRS, ELSA, and CHARLS.

Characteristics	HRS		*P*-value	ELSA		*P*-value	CHARLS		*P*-value
	Non-frail	Frail		Non-frail	Frail		Non-frail	Frail	
**Number (%)**	2038	655		5323	1056		1732	634	
**eCRF, mean (SD)**	9.35 (2.11)	8.37 (2.18)	<0.001	9.16 (1.84)	7.73 (1.85)	<0.001	10.73 (1.94)	9.82 (2.03)	<0.001
**Age, mean (SD), years**	71.68 (5.04)	72.67 (5.45)	<0.001	64.02 (7.88)	67.15 (8.29)	<0.001	60.38 (7.02)	63.14 (7.41)	<0.001
**Sex, *n* (%)**			0.093			<0.001			<0.001
Male	1028 (50.44%)	305 (46.56%)		2614 (49.11%)	451 (42.71%)		1061 (61.26%)	299 (47.16%)	
Female	1010 (49.56%)	350 (53.44%)		2709 (50.89%)	605 (57.29%)		671 (38.74%)	335 (52.84%)	
**BMI, mean (SD), kg/m²**	28.30 (4.77)	30.57 (6.15)	<0.001	27.69 (4.48)	29.90 (5.46)	<0.001	23.56 (3.42)	24.09 (3.44)	<0.001
**WC, mean (SD), cm**	39.10 (5.12)	41.64 (5.86)	<0.001	95.19 (12.57)	100.64 (13.58)	<0.001	84.71 (12.01)	86.81 (13.48)	<0.001
**rHR, mean (SD), bpm**	69.32 (10.94)	70.35 (12.30)	0.057	66.97 (10.72)	70.41 (12.10)	<0.001	71.72 (10.38)	72.63 (11.30)	0.077
**Physical activity, *n* (%)**			<0.001			<0.001			<0.001
Others	575 (28.21%)	383 (58.47%)		1391 (26.13%)	691 (65.44%)		545 (31.47%)	265 (41.80%)	
Moderate-to-vigorous	1463 (71.79%)	272 (41.53%)		3932 (73.87%)	365 (34.56%)		1187 (68.53%)	369 (58.20%)	
**Smoking status, *n* (%)**			0.007			<0.001			<0.001

Characteristics	HRS		*P*-value	ELSA		*P*-value	CHARLS		*P*-value
	Non-frail	Frail		Non-frail	Frail		Non-frail	Frail	
Never	861 (42.25%)	237 (36.18%)		2044 (38.40%)	295 (27.94%)		885 (51.10%)	377 (59.46%)	
Former	976 (47.89%)	333 (50.84%)		2570 (48.28%)	523 (49.53%)		188 (10.85%)	82 (12.93%)	
Current	201 (9.86%)	85 (12.98%)		709 (13.32%)	237 (22.44%)		659 (38.05%)	175 (27.60%)	

Abbreviations: HRS, Health and Retirement Study; ELSA, English Longitudinal Study of Ageing; CHARLS, China Health and Retirement Longitudinal Study; eCRF, estimated cardiorespiratory fitness; BMI, body mass index; WC, waist circumference; rHR, resting heart rate.

Table S6. Baseline characteristics of HRS participants stratified by eCRF groups based on quintiles.

Characteristics	Overall	Low eCRF	Medium eCRF	High eCRF	*P*-value
**Number (%)**	1900	272	748	880	
**eCRF, mean (SD)**	9.33 (2.10)	7.42 (1.71)	8.99 (1.81)	10.21 (1.96)	<0.001
**Age, mean (SD), years**	71.54 (4.87)	71.35 (4.50)	71.60 (4.77)	71.54 (5.06)	0.757
**Sex, *n* (%)**					0.324
Male	938 (49.37%)	135 (49.63%)	384 (51.34%)	419 (47.61%)	
Female	962 (50.63%)	137 (50.37%)	364 (48.66%)	461 (52.39%)	
**Education, *n* (%)**					0.001
Below high school	299 (15.74%)	52 (19.12%)	130 (17.38%)	117 (13.30%)	
High school	722 (38.00%)	107 (39.34%)	303 (40.51%)	312 (35.45%)	
Above high school	879 (46.26%)	113 (41.54%)	315 (42.11%)	451 (51.25%)	
**Employment, *n* (%)**					0.532
Unemployed	132 (6.95%)	18 (6.62%)	58 (7.75%)	56 (6.36%)	
Working or retired	1768 (93.05%)	254 (93.38%)	690 (92.25%)	824 (93.64%)	
**Marital status, *n* (%)**					0.031
Unmarried and others	513 (27.00%)	88 (32.35%)	209 (27.94%)	216 (24.55%)	
Married and partnered	1387 (73.00%)	184 (67.65%)	539 (72.06%)	664 (75.45%)	
**Co-residence with children, *n* (%)**					0.001
No	1555 (81.84%)	207 (76.10%)	600 (80.21%)	748 (85.00%)	

Characteristics	Overall	Low eCRF	Medium eCRF	High eCRF	*P*-value
Yes	345 (18.16%)	65 (23.90%)	148 (19.79%)	132 (15.00%)	
**Smoking status, *n* (%)**					0.063
Never	812 (42.74%)	117 (43.01%)	294 (39.30%)	401 (45.57%)	
Former	902 (47.47%)	130 (47.79%)	367 (49.06%)	405 (46.02%)	
Current	186 (9.79%)	25 (9.19%)	87 (11.63%)	74 (8.41%)	
**Alcohol consumption, *n* (%)**					<0.001
No	788 (41.47%)	132 (48.53%)	331 (44.25%)	325 (36.93%)	
Yes	1112 (58.53%)	140 (51.47%)	417 (55.75%)	555 (63.07%)	
**Physical activity, *n* (%)**					<0.001
Others	530 (27.89%)	148 (54.41%)	257 (34.36%)	125 (14.20%)	
Moderate-to-vigorous	1370 (72.11%)	124 (45.59%)	491 (65.64%)	755 (85.80%)	
**BMI, mean (SD), kg/m²**	28.37 (4.76)	35.87 (3.85)	29.62 (2.83)	24.99 (2.72)	<0.001
**WC, mean (SD), cm**	39.13 (5.13)	45.61 (4.15)	40.55 (3.70)	35.92 (3.85)	<0.001
**rHR, mean (SD), bpm**	69.22 (10.70)	75.64 (11.06)	71.80 (10.26)	65.04 (9.19)	<0.001
**Cancer, *n* (%)**	273 (14.37%)	35 (12.87%)	103 (13.77%)	135 (15.34%)	0.499
**Lung disease, *n* (%)**	99 (5.21%)	16 (5.88%)	44 (5.88%)	39 (4.43%)	0.366
**CVD, *n* (%)**	411 (21.63%)	52 (19.12%)	146 (19.52%)	213 (24.20%)	0.040
**FI, median (IQR)**	0.12 (0.08, 0.18)	0.15 (0.11, 0.21)	0.12 (0.08, 0.18)	0.11 (0.06, 0.17)	<0.001
**Follow-up time, mean (SD), years**	8.14 (4.18)	7.41 (3.95)	7.88 (4.18)	8.59 (4.21)	<0.001

Abbreviations: SD standard deviation, HRS Health and Retirement Study, eCRF estimated cardiorespiratory fitness, BMI body mass index, WC waist circumference, rHR resting heart rate, FI frailty index.

Bold text is used to indicate subgroup categories for clarity.

Table S7. Baseline characteristics of ELSA participants stratified by eCRF groups based on quintiles.

**Characteristics**	**Overall**	**Low eCRF**	**Medium eCRF**	**High eCRF**	***P*-value**
**Number (%)**	4816	734	1920	2162	
**eCRF, mean (SD)**	9.19 (1.85)	7.25 (1.40)	8.81 (1.45)	10.18 (1.65)	<0.001
**Age, mean (SD), years**	63.87 (7.83)	63.24 (7.71)	63.97 (7.84)	63.99 (7.87)	0.064
**Sex, *n* (%)**					0.508
Male	2347 (48.73%)	371 (50.54%)	922 (48.02%)	1054 (48.75%)	
Female	2469 (51.27%)	363 (49.46%)	998 (51.98%)	1108 (51.25%)	
**Education, *n* (%)**					<0.001
Below high school	1669 (37.91%)	311 (46.14%)	700 (40.02%)	658 (33.25%)	
High school	946 (21.49%)	134 (19.88%)	376 (21.50%)	436 (22.03%)	
Above high school	1787 (40.60%)	229 (33.98%)	673 (38.48%)	885 (44.72%)	
**Employment, *n* (%)**					0.888
Unemployed	42 (2.15%)	8 (2.52%)	16 (2.07%)	18 (2.09%)	
Working or retired	1908 (97.85%)	310 (97.48%)	756 (97.93%)	842 (97.91%)	
**Marital status, *n* (%)**					0.592
Unmarried and others	1171 (24.32%)	189 (25.75%)	458 (23.85%)	524 (24.25%)	
Married and partnered	3644 (75.68%)	545 (74.25%)	1462 (76.15%)	1637 (75.75%)	
**Co-residence with children, *n* (%)**					0.084
No	3196 (77.20%)	475 (73.87%)	1288 (77.50%)	1433 (78.09%)	

Characteristics	Overall	Low eCRF	Medium eCRF	High eCRF	*P*-value
Yes	944 (22.80%)	168 (26.13%)	374 (22.50%)	402 (21.91%)	
**Smoking status, *n* (%)**					<0.001
Never	1879 (39.02%)	237 (32.29%)	728 (37.92%)	914 (42.28%)	
Former	2310 (47.97%)	371 (50.54%)	907 (47.24%)	1032 (47.73%)	
Current	627 (13.02%)	126 (17.17%)	285 (14.84%)	216 (9.99%)	
**Alcohol consumption, *n* (%)**					0.032
No	316 (7.06%)	61 (9.21%)	128 (7.20%)	127 (6.23%)	
Yes	4162 (92.94%)	601 (90.79%)	1649 (92.80%)	1912 (93.77%)	
**Physical activity, *n* (%)**					<0.001
Others	1226 (25.46%)	378 (51.50%)	586 (30.52%)	262 (12.12%)	
Moderate-to-vigorous	3590 (74.54%)	356 (48.50%)	1334 (69.48%)	1900 (87.88%)	
**BMI, mean (SD), kg/m²**	27.66 (4.48)	34.51 (4.28)	28.62 (2.62)	24.48 (2.36)	<0.001
**WC, mean (SD), cm**	95.05 (12.52)	110.92 (10.80)	97.88 (8.99)	87.14 (9.09)	<0.001
**rHR, mean (SD), bpm**	66.87 (10.65)	73.82 (11.20)	68.93 (10.24)	62.68 (8.90)	<0.001
**Cancer, *n* (%)**	288 (5.98%)	42 (5.72%)	115 (5.99%)	131 (6.06%)	0.946
**Lung disease, *n* (%)**	171 (3.55%)	24 (3.27%)	70 (3.65%)	77 (3.56%)	0.896
**CVD, *n* (%)**	634 (13.16%)	81 (11.04%)	269 (14.01%)	284 (13.14%)	0.128
**FI, median (IQR)**	0.08 (0.04, 0.14)	0.11 (0.05, 0.16)	0.08 (0.04, 0.14)	0.07 (0.04, 0.11)	<0.001
**Follow-up time, mean (SD), years**	9.57 (4.62)	8.48 (4.71)	9.31 (4.67)	10.18 (4.45)	<0.001

Abbreviations: SD standard deviation, ELSA English Longitudinal Study of Ageing, eCRF estimated cardiorespiratory fitness, BMI body mass index, WC waist circumference, rHR resting heart rate, FI frailty index.

Bold text is used to indicate subgroup categories for clarity.

Table S8. Baseline characteristics of CHARLS participants stratified by eCRF groups based on quintiles.

**Characteristics**	**Overall**	**Low eCRF**	**Medium eCRF**	**High eCRF**	***P*-value**
**Number (%)**	1436	266	625	545	
**eCRF, mean (SD)**	10.78 (1.94)	9.34 (1.61)	10.54 (1.69)	11.77 (1.82)	<0.001
**Age, mean (SD), years**	60.28 (6.88)	60.51 (6.98)	60.53 (7.01)	59.88 (6.67)	0.227
**Sex, *n* (%)**					0.080
Male	885 (61.63%)	180 (67.67%)	376 (60.16%)	329 (60.37%)	
Female	551 (38.37%)	86 (32.33%)	249 (39.84%)	216 (39.63%)	
**Education, *n* (%)**					0.002
Below high school	939 (65.39%)	148 (55.64%)	415 (66.40%)	376 (68.99%)	
High school	429 (29.87%)	104 (39.10%)	176 (28.16%)	149 (27.34%)	
Above high school	68 (4.74%)	14 (5.26%)	34 (5.44%)	20 (3.67%)	
**Employment, *n* (%)**					0.085
Unemployed	19 (1.33%)	7 (2.64%)	8 (1.28%)	4 (0.74%)	
Working or retired	1410 (98.67%)	258 (97.36%)	615 (98.72%)	537 (99.26%)	
**Marital status, *n* (%)**					0.336
Unmarried and others	142 (9.89%)	24 (9.02%)	56 (8.96%)	62 (11.38%)	
Married and partnered	1294 (90.11%)	242 (90.98%)	569 (91.04%)	483 (88.62%)	
**Co-residence with children, *n* (%)**					0.913
No	622 (44.37%)	117 (44.66%)	275 (44.86%)	230 (43.64%)	

Characteristics	Overall	Low eCRF	Medium eCRF	High eCRF	*P*-value
Yes	780 (55.63%)	145 (55.34%)	338 (55.14%)	297 (56.36%)	
**Smoking status, *n* (%)**					0.292
Never	733 (51.04%)	123 (46.24%)	318 (50.88%)	292 (53.58%)	
Former	162 (11.28%)	36 (13.53%)	73 (11.68%)	53 (9.72%)	
Current	541 (37.67%)	107 (40.23%)	234 (37.44%)	200 (36.70%)	
**Alcohol consumption, *n* (%)**					0.905
No	627 (43.69%)	113 (42.48%)	275 (44.07%)	239 (43.85%)	
Yes	808 (56.31%)	153 (57.52%)	349 (55.93%)	306 (56.15%)	
**Physical activity, *n* (%)**					<0.001
Others	434 (30.22%)	162 (60.90%)	210 (33.60%)	62 (11.38%)	
Moderate-to-vigorous	1002 (69.78%)	104 (39.10%)	415 (66.40%)	483 (88.62%)	
**BMI, mean (SD), kg/m²**	23.54 (3.41)	27.75 (3.90)	23.77 (2.33)	21.22 (1.69)	<0.001
**WC, mean (SD), cm**	84.53 (12.06)	95.77 (9.86)	86.16 (8.27)	77.17 (11.79)	<0.001
**rHR, mean (SD), bpm**	71.60 (10.23)	78.19 (10.34)	73.33 (9.42)	66.39 (8.39)	<0.001
**Cancer, *n* (%)**	11 (0.77%)	1 (0.38%)	6 (0.96%)	4 (0.73%)	0.654
**Lung disease, *n* (%)**	103 (7.17%)	21 (7.89%)	49 (7.84%)	33 (6.06%)	0.439
**CVD, *n* (%)**	140 (9.75%)	32 (12.03%)	74 (11.84%)	34 (6.24%)	0.002
**FI, median (IQR)**	0.13 (0.08, 0.18)	0.15 (0.11, 0.19)	0.13 (0.08, 0.18)	0.12 (0.08, 0.18)	0.013
**Follow-up time, mean (SD), years**	4.90 (2.03)	4.52 (2.07)	4.91 (2.02)	5.08 (2.00)	<0.001

Abbreviations: SD standard deviation, CHARLS China Health and Retirement Longitudinal Study, eCRF estimated cardiorespiratory fitness, BMI body mass index, WC waist circumference, rHR resting heart rate, FI frailty index.

Bold text is used to indicate subgroup categories for clarity.

Table S9. Schoenfeld residuals test for the Cox proportional hazards assumption in HRS.

**Variables**	χ^2	**df**	***P*-value**
eCRF	2.55	2	0.279
Age	0.88	1	0.349
Sex	0.26	1	0.611
Education	3.47	2	0.176
Employment	3.14	1	0.076
Marital Status	2.81	1	0.094
Co-residence with children	0.03	1	0.872
Alcohol consumption	0.23	1	0.631
Global test	12.09	10	0.279

Abbreviations: HRS, Health and Retirement Study; eCRF, estimated cardiorespiratory fitness.

Table S10. Schoenfeld residuals test for the Cox proportional hazards assumption in ELSA.

**Variables**	χ^2	**df**	***P*-value**
eCRF	3.30	2	0.192
Age	0.02	1	0.886
Sex	0.03	1	0.873
Education	3.33	2	0.189
Employment	<0.01	1	0.996
Marital Status	5.80	1	0.016
Co-residence with children	<0.01	1	0.973
Alcohol consumption	0.47	1	0.494
Global test	13.14	10	0.216

Abbreviations: ELSA, English Longitudinal Study of Ageing; eCRF, estimated cardiorespiratory fitness.

Table S11. Schoenfeld residuals test for the Cox proportional hazards assumption in CHARLS.

**Variables**	**χ^2**	**df**	***P*-value**
eCRF	2.65	2	0.266
Age	0.12	1	0.724
Sex	0.19	1	0.665
Education	4.79	2	0.091
Employment	<0.01	1	0.996
Marital Status	0.75	1	0.387
Co-residence with children	0.22	1	0.638
Alcohol consumption	0.25	1	0.618
Global test	8.10	10	0.619

Abbreviations: CHARLS, China Health and Retirement Longitudinal Study; eCRF, estimated cardiorespiratory fitness.

Table S12. Association of eCRF with risks of incident frailty after excluding participants with incident frailty during the first wave of follow-up.

	HRS			ELSA			CHARLS		
**eCRF, METs**	**Events/n**	**HR (95% CI)**	***P*-value**	**Events/n**	**HR (95% CI)**	***P*-value**	**Events/n**	**HR (95% CI)**	***P*-value**
eCRF per 1-SD	692/1619	0.58 (0.51-0.67)	<0.001	1028/4120	0.54 (0.45-0.66)	<0.001	312/1118	0.82 (0.66-1.01)	0.063
Low eCRF level	142/229	reference		220/587	reference		61/187	reference	
Moderate eCRF level	277/628	0.62 (0.50-0.76)	<0.001	437/1615	0.71 (0.50-1.01)	0.059	135/490	0.71 (0.52-0.96)	0.028
High eCRF level	273/762	0.47 (0.38-0.58)	<0.001	371/1918	0.34 (0.23-0.50)	<0.001	116/441	0.67 (0.49-0.92)	0.012
p for trend			<0.001			<0.001			0.053

Abbreviations: HRS, Health and Retirement Study; ELSA, English Longitudinal Study of Ageing; CHARLS, China Health and Retirement Longitudinal Study; eCRF, estimated cardiorespiratory fitness; HR, hazard ratio; CI, confidence interval.

The model adjusted age, sex, education, employment status, marital status, co-residence with children, and alcohol consumption.

Table S13. Association of eCRF with risks of incident frailty after excluding participants with cancer, lung diseases or cardiovascular disease at baseline.

	HRS			ELSA			CHARLS		
**eCRF, METs**	**Events/n**	**HR (95% CI)**	***P*-value**	**Events/n**	**HR (95% CI)**	***P*-value**	**Events/n**	**HR (95% CI)**	***P*-value**
eCRF per 1-SD	487/1230	0.52 (0.45-0.61)	<0.001	927/3812	0.55 (0.45-0.67)	<0.001	367/1192	0.77 (0.64-0.93)	0.007
Low eCRF level	110/183	reference		220/600	reference		81/214	reference	
Moderate eCRF level	205/495	0.64 (0.51-0.81)	<0.001	400/1498	0.64 (0.44-0.92)	0.016	153/504	0.63 (0.48-0.83)	0.001
High eCRF level	172/552	0.43 (0.34-0.55)	<0.001	307/1714	0.31 (0.20-0.46)	<0.001	133/474	0.57 (0.43-0.76)	<0.001
p for trend			<0.001			<0.001			<0.001

Abbreviations: HRS, Health and Retirement Study; ELSA, English Longitudinal Study of Ageing; CHARLS, China Health and Retirement Longitudinal Study; eCRF, estimated cardiorespiratory fitness; HR, hazard ratio; CI, confidence interval.

The model adjusted age, sex, education, employment status, marital status, co-residence with children, and alcohol consumption.

Table S14. Association of eCRF with risks of incident frailty after excluding participants missing important covariates at baseline.

	HRS			ELSA			CHARLS		
**eCRF, METs**	**Events/n**	**HR (95% CI)**	***P*-value**	**Events/n**	**HR (95% CI)**	***P*-value**	**Events/n**	**HR (95% CI)**	***P*-value**
eCRF per 1-SD	871/1900	0.60 (0.54-0.68)	<0.001	230/1435	0.56 (0.48-0.66)	<0.001	479/1394	0.74 (0.63-0.87)	<0.001
Low eCRF level	175/272	reference		61/224	reference		110/261	reference	
Moderate eCRF level	360/748	0.67 (0.56-0.81)	<0.001	111/575	0.66 (0.48-0.91)	0.010	208/610	0.64 (0.50-0.80)	<0.001
High eCRF level	336/880	0.50 (0.41-0.60)	<0.001	58/636	0.30 (0.21-0.43)	<0.001	161/523	0.56 (0.44-0.71)	<0.001
p for trend			<0.001			<0.001			<0.001

Abbreviations: HRS, Health and Retirement Study; ELSA, English Longitudinal Study of Ageing; CHARLS, China Health and Retirement Longitudinal Study; eCRF, estimated cardiorespiratory fitness; HR, hazard ratio; CI, confidence interval.

The model adjusted age, sex, education, employment status, marital status, co-residence with children, and alcohol consumption.

Table S15. Association of eCRF with risks of incident frailty with multiple imputation.

	HRS			ELSA			CHARLS		
**eCRF, METs**	**Events/n**	**HR (95% CI)**	***P*-value**	**Events/n**	**HR (95% CI)**	***P*-value**	**Events/n**	**HR (95% CI)**	***P*-value**
eCRF per 1-SD	871/1900	0.60 (0.54-0.68)	<0.001	1328/4816	0.59 (0.54-0.64)	<0.001	489/1436	0.72 (0.62-0.84)	<0.001
Low eCRF level	175/272	reference		299/734	reference		113/266	reference	
Moderate eCRF level	360/748	0.67 (0.56-0.81)	<0.001	580/1920	0.63 (0.55-0.73)	<0.001	210/625	0.64 (0.50-0.80)	<0.001
High eCRF level	336/880	0.50 (0.41-0.60)	<0.001	449/2162	0.39 (0.34-0.46)	<0.001	166/545	0.56 (0.44-0.71)	<0.001
p for trend			<0.001			<0.001			<0.001

Abbreviations: HRS, Health and Retirement Study; ELSA, English Longitudinal Study of Ageing; CHARLS, China Health and Retirement Longitudinal Study; eCRF, estimated cardiorespiratory fitness; HR, hazard ratio; CI, confidence interval.

The model adjusted age, sex, education, employment status, marital status, co-residence with children, and alcohol consumption.

Table S16. Association of eCRF with risks of incident frailty after additional adjustment for baseline FI.

Characteristics	Events/n	Model1		Model2		Model3	
		HR (95% CI)	*P*-value	HR (95% CI)	*P*-value	HR (95% CI)	*P*-value
HRS							
eCRF per 1-SD	871/1900	0.79 (0.74-0.85)	<0.001	0.58 (0.52-0.65)	<0.001	0.84 (0.75-0.95)	0.006
Low eCRF level	175/272	reference		reference		reference	
Moderate eCRF level	360/748	0.68 (0.56-0.81)	<0.001	0.66 (0.55-0.79)	<0.001	0.85 (0.71-1.02)	0.085
High eCRF level	336/880	0.48 (0.40-0.58)	<0.001	0.47 (0.39-0.56)	<0.001	0.78 (0.65-0.95)	0.011
P for trend			<0.001		<0.001		0.014
ELSA							
eCRF per 1-SD	1328/4816	0.53 (0.50-0.56)	<0.001	0.56 (0.52-0.61)	<0.001	0.68 (0.57-0.82)	<0.001
Low eCRF level	299/734	reference		reference		reference	
Moderate eCRF level	580/1920	0.66 (0.58-0.76)	<0.001	0.60 (0.52-0.69)	<0.001	0.75 (0.55-1.03)	0.077
High eCRF level	449/2162	0.41 (0.35-0.48)	<0.001	0.37 (0.32-0.42)	<0.001	0.46 (0.32-0.67)	<0.001
P for trend			<0.001		<0.001		<0.001

Characteristics	Events/n	Model1		Model2		Model3	
		HR (95% CI)	*P*-value	HR (95% CI)	*P*-value	HR (95% CI)	*P*-value
CHARLS							
eCRF per 1-SD	489/1436	0.72 (0.66-0.79)	<0.001	0.75 (0.64-0.88)	<0.001	0.80 (0.68-0.94)	0.005
Low eCRF level	113/266	reference		reference		reference	
Moderate eCRF level	210/625	0.70 (0.56-0.88)	0.002	0.66 (0.52-0.83)	<0.001	0.69 (0.55-0.87)	0.002
High eCRF level	166/545	0.61 (0.48-0.77)	<0.001	0.59 (0.46-0.75)	<0.001	0.63 (0.50-0.81)	<0.001
P for trend			<0.001		<0.001		<0.001

Abbreviations: HRS, Health and Retirement Study; ELSA, English Longitudinal Study of Ageing; CHARLS, China Health and Retirement Longitudinal Study; eCRF, estimated cardiorespiratory fitness; HR, hazard ratio; CI, confidence interval.

Model 1: Unadjusted

Model 2: Adjusted for age and sex

Model 3: Fully adjusted for age, sex, education, employment status, marital status, co-residence with children, alcohol consumption, and baseline FI
